# Inactivation Efficacy of Nonthermal Plasma-Activated Solutions against Newcastle Disease Virus

**DOI:** 10.1128/AEM.02836-17

**Published:** 2018-04-16

**Authors:** Xia Su, Ying Tian, Hongzhuan Zhou, Yinglong Li, Zhenhua Zhang, Beiyu Jiang, Bing Yang, Jue Zhang, Jing Fang

**Affiliations:** a Beijing Key Laboratory for Prevention and Control of Infectious Diseases in Livestock and Poultry, Institute of Animal Husbandry and Veterinary Medicine, Beijing Academy of Agriculture and Forestry Sciences, Beijing, People's Republic of China; b Academy for Advanced Interdisciplinary Studies, Peking University, Beijing, People's Republic of China; c College of Engineering, Peking University, Beijing, People's Republic of China; Rutgers, The State University of New Jersey

**Keywords:** Newcastle disease virus, plasma-activated solution, inactivation efficacy, biological damage, physicochemical property

## Abstract

In recent years, plasma-activated solutions (PASs) have made good progress in the disinfection of medical devices, tooth whitening, and fruit preservation. In this study, we investigated the inactivation efficacy of Newcastle disease virus by PASs. Water, 0.9% NaCl, and 0.3% H_2_O_2_ were excited by plasma to obtain the corresponding solutions PAS(H_2_O), PAS(NaCl), and PAS(H_2_O_2_). The complete inactivation of virus after PAS treatment for 30 min was confirmed by the embryo lethality assay (ELA) and hemagglutination (HA) test. Scanning electron microscopy (SEM) results showed that the morphology of the viral particle changed under PAS treatments. The total protein concentration of virus decreased as measured by a Bradford protein assay due to PAS treatment. The nucleic acid integrity assay demonstrated that viral RNA degraded into smaller fragments. Moreover, the physicochemical properties of PASs, including the oxidation-reduction potential (ORP), electrical conductivity, and H_2_O_2_ concentration, and electron spin resonance spectra analysis indicated that reactive oxygen and nitrogen species play a major role in the virus inactivation. Therefore, the application of PASs, as an environmentally friendly method, would be a promising alternative strategy in poultry industries.

**IMPORTANCE** Newcastle disease (ND), as an infectious viral disease of avian species, caused significant economic losses to domestic animal and poultry industries. The traditional chemical sanitizers, such as chlorine-based products, are associated with risks of by-product formation with carcinogenic effects and environmental pollution. On the basis of this, plasma-activated water as a green disinfection product is a promising alternative for applications in stock farming and sterilization in hospitals and public places. In this study, we explored the inactivation efficacy of different plasma-activated solutions (PASs) against ND virus (NDV) and the possible underlying mechanisms. Our results demonstrated that reactive oxygen and nitrogen species detected in PASs, including short-lived OH˙ and NO˙ and long-lived H_2_O_2_, changed the morphology, destroyed the RNA structure, and degraded the protein of the virus, consequently resulting in virus inactivation. These lay a foundation for the application of PASs to resolve the issues of public health and environmental sanitation.

## INTRODUCTION

Newcastle disease (ND) is a highly infectious viral disease of avian species, which can cause significant economic losses to domestic animal and poultry industries (1, 2). The influence of ND is most remarkable in poultry, which might result from the high susceptibility of poultry and the grave consequences of successive outbreaks of virulent strains in different parts of the world (3). From the perspective of the loss of livestock, ND ranked as the fourth important disease (4). Moreover, the number of bird species infected by ND virus (NDV) has reached 236. In addition to poultry species, virulent NDV isolates are usually found in double-crested cormorants and pigeons (5–7).

The virus strains of low virulence may result in mild enteric infections. Viruses of intermediate virulence primarily bring about respiratory disease, while virulent viruses may lead to 100% mortality (8). The clinical signs observed with an NDV infection will be nonspecific, including open mouth breathing, depression, ruffled feathers, anorexia, and listlessness before death (9). Furthermore, NDV is also pathogenic to humans (10). The most commonly reported clinical symptoms in human infections are eye infections, usually accompanied by unilateral or bilateral reddening, excessive lacrimation, and eyelid or conjunctival congestion. Other infection signs may sometimes occur, causing chills, headaches, and fever. Therefore, it is necessary to develop antiviral agents that can effectively prevent the spread of viruses and eliminate viruses from the environment.

Nonthermal plasma is a complex mixture, which consists of charged particles, reactive species, and UV photons (11). It has been proved effective in biomedical fields, including for decontamination and sterilization (12, 13), teeth whitening (14), and food preservation (15, 16). Plasma-activated water is prepared by treating distilled water with nonthermal plasma and has been considered a potential disinfectant against a variety of microbes (17–20). It is generally accepted that the disinfection efficacy of plasma-activated water depends on the synergistic effects of reactive oxygen and nitrogen species (RONS) such as the hydroxyl radical (OH˙), atomic oxygen (O), hydrogen peroxide (H_2_O_2_), and nitric oxide (NO˙) and its derivatives formed with water, including nitrites (NO_2_^−^), nitrates (NO_3_^−^), and peroxynitrites (ONOOH) (21–25). As a “green” disinfection product, plasma-activated water is a promising alternative to traditional sanitizers applied in agriculture and for sterilization in hospitals and public places. Recent studies have reported that plasma-activated water could inactivate foodborne microbes on fruits and maintain the postharvest quality of mushrooms (15, 16). It was also proposed that the plasma-activated medium could kill cancer cells in a dose-dependent manner (26). In addition, the treatment of water with nonthermal plasma discharges could contribute to the change of its physicochemical properties, which in turn may influence the plant growth and agriculture produce quality (27). To our knowledge, a few research studies have verified the inactivation effect of cold plasma on viruses (28–31). However, there is no information on the inactivation effect of plasma-activated water and the mechanism of interaction, which are well worth investigating.

In this study, three different solutions (H_2_O, 0.9% NaCl, and 0.3% H_2_O_2_) were excited by nonthermal plasma to obtain the corresponding plasma-activated solutions (PASs), namely, PAS(H_2_O), PAS(NaCl), and PAS(H_2_O_2_). The inactivation efficacies of the PASs on NDV were investigated by the embryo lethality assay (ELA) and the hemagglutination (HA) test. Scanning electron microscopy (SEM) was used to observe the morphological changes of the viruses after PAS treatment. Furthermore, the biological properties of the virus, including proteins and nucleic acids, were analyzed. Additionally, the physicochemical characteristics of the PASs, including the oxidation-reduction potential (ORP), electrical conductivity, and the concentrations of H_2_O_2_ and some other short-lived species were measured.

## RESULTS

### Inactivation efficacy of PAS.

**FIG 1:**
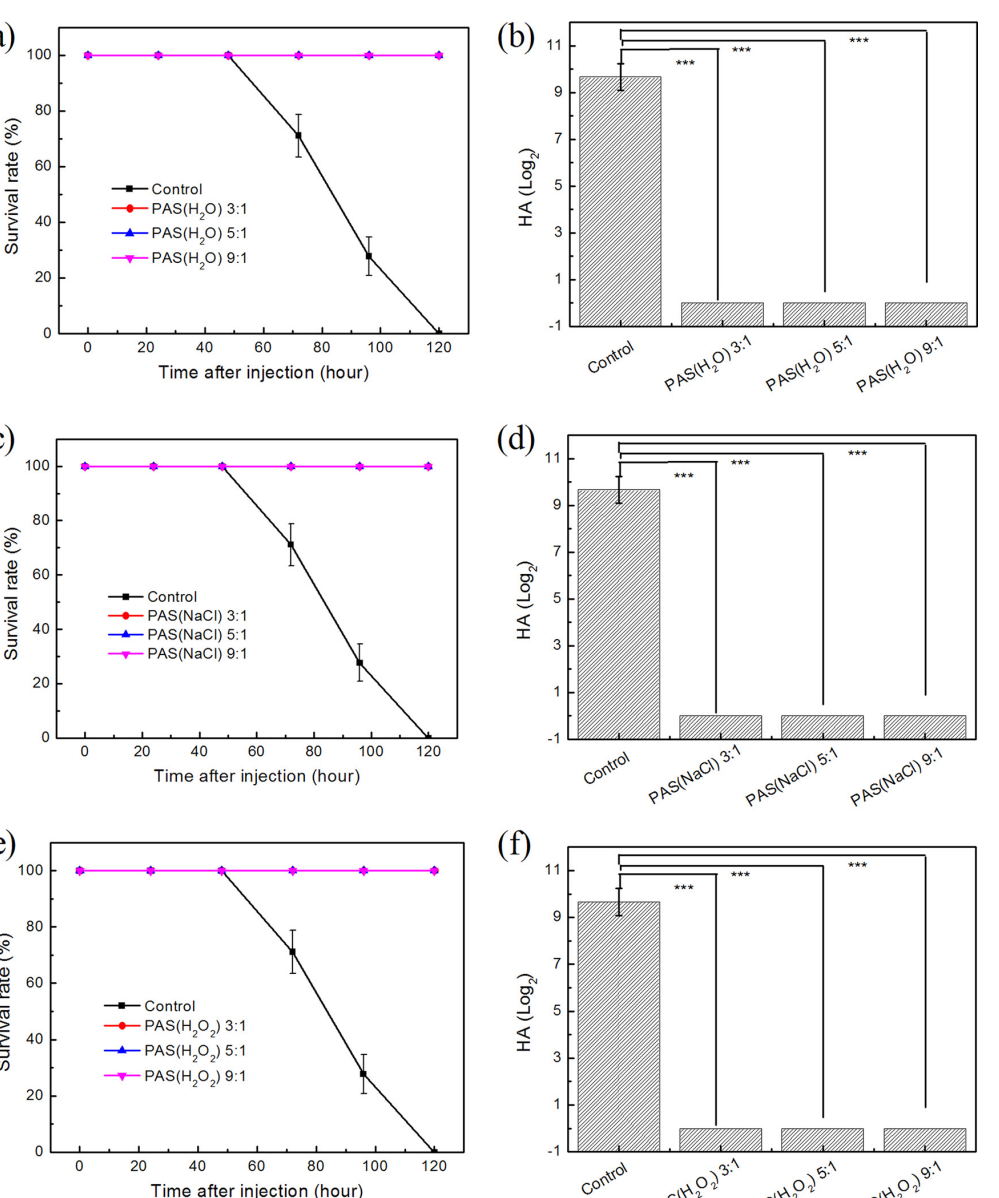
Inactivation efficacy of PASs. (a, c, and e) The survival rate curves of embryos for 120 h after injections of PAS(H_2_O)-treated, PAS(NaCl)-treated, and PAS(H_2_O_2_)-treated NDV. (b, d, and f) The HA titers of allantoic fluids extracted from embryos inoculated with PAS(H_2_O)-treated, PAS(NaCl)-treated, and PAS(H_2_O_2_)-treated NDV. ***, *P* < 0.001 between the PAS groups and the control group.

In Fig. 1, the inactivation efficacies of PAS(H_2_O), PAS(NaCl), and PAS(H_2_O_2_) were verified. The ELA results are shown in Fig. 1a, c, and e. In the control group, the survival rate of chicken embryos was 71.13% ± 7.68% at 72 h and decreased to 27.77% ± 6.93% at 96 h. After 120 h of inoculation, all chicken embryos were dead. However, the survival rates of chicken embryos treated with PAS(H_2_O), PAS(NaCl), and PAS(H_2_O_2_) were 100% when the PAS and NDV interacted at ratios of 3:1, 5:1, and 9:1.

Correspondingly, Fig. 1b, d, and f show the results of the hemagglutination test, and the virus titers in the allantoic fluids of chicken embryos after 120 h of inoculation were examined. Compared to the control group with an average HA titer of 9.40 (± 0.58) log_2_, no HA titers of any of the treatment groups were detected.

To further assess whether NDV was inactivated completely after PAS treatment, ELAs and HA tests were used to record the survival of chicken embryos and test the virulence of allantoic fluids after three passages. According to Table 1, the chicken embryos in the treatment group all survived after serial passages, while the chicken embryos in the control group died. Correspondingly, there was no change in the virus titer, which was still undetected, in the allantoic fluids of chicken embryos.

**TABLE 1:** Virulence assessment of NDV in chicken embryos after PAS treatment

Treatment	ELA (no./total no. of embryos)^*a*^			HA titer (reciprocal log_2_)^*b*^		
	F1	F2	F3	F1	F2	F3
Control	0/5	0/5	0/5	9	9	9
PAS(H_2_O)	5/5	5/5	5/5	0	0	0
PAS(NaCl)	5/5	5/5	5/5	0	0	0
PAS(H_2_O_2_)	5/5	5/5	5/5	0	0	0

a Numbers of chicken embryos surviving 120 h after injections of PAS(H_2_O)-treated, PAS(NaCl)-treated, and PAS(H_2_O_2_)-treated NDV.

b Mean virus titers of allantoic fluid from responding chickens.

### Physicochemical properties of PAS.

**FIG 2:**
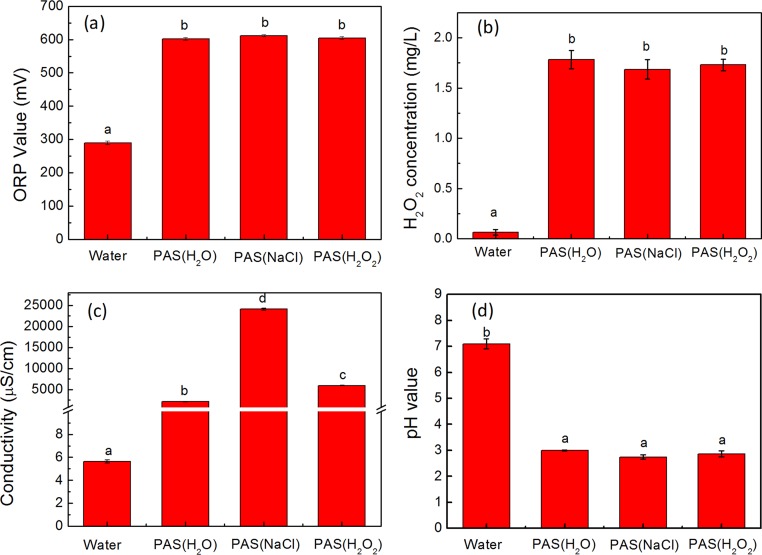
Physicochemical properties of PASs. ORP values (a), H_2_O_2_ concentrations (b), electrical conductivity (c), and pH values (d) of PAS(H_2_O), PAS(NaCl), and PAS(H_2_O_2_). Bars labeled with different lowercase letters represent significant differences (*P* ≤ 0.05).

The ORP value reflects the general oxidation capacity of the solution. In this study, ORP values were measured immediately after PAS generation. As shown in Fig. 2a, the ORP of deionized water was only approximately 290 mV, while the ORP values of PAS(H_2_O), PAS(NaCl), and PAS(H_2_O_2_) significantly increased, reaching 602 mV, 612 mV, and 604 mV, respectively (*P* < 0.05). Moreover, no significant differences were seen among PAS(H_2_O), PAS(NaCl), and PAS(H_2_O_2_) groups.

H_2_O_2_, as a major long-lived species, was examined in PAS(H_2_O), PAS (NaCl), and PAS (H_2_O_2_). The concentration of H_2_O_2_ in water was only 0.05 mg/liter as shown in Fig. 2b. After plasma treatment, the concentrations of H_2_O_2_ in PAS(H_2_O), PAS (NaCl), and PAS (H_2_O_2_) were significantly increased, achieving 1.60, 1.40, and 1.50 mg/liter, respectively. There was no significant difference among the different PASs.

In addition, the conductivities of PAS(H_2_O), PAS(NaCl), and PAS(H_2_O_2_) were recorded in the experiment. As shown in Fig. 2c, compared to the conductivity of water, which was only 5.60 μS/cm, the conductivity after plasma activation was significantly higher, achieving 2.10, 24.10, and 6.0 mS/cm for PAS(H_2_O), PAS(NaCl), and PAS(H_2_O_2_), respectively. Furthermore, the conductivity of PAS(NaCl) was higher than those of PAS(H_2_O) and PAS(H_2_O_2_).

The pH values of the PASs were also recorded during the experiments. The initial pH value of the sterile water was around 7.1. As presented in Fig. 2d, the pH of PAS(H_2_O), PAS(NaCl), and PAS(H_2_O_2_) decreased to 3.0, 2.7, and 2.9, respectively, after plasma activation. There were significant differences between the water group and PAS groups.

### Evaluation of NO radical and OH radical.

**FIG 3:**
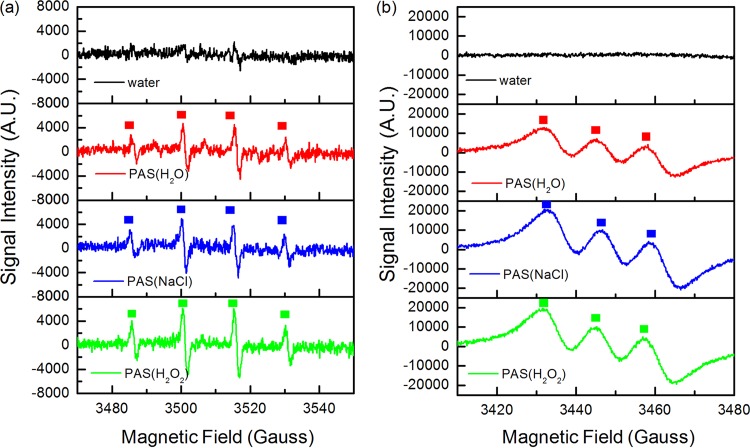
Electron spin resonance spin-trapping spectra of DMPO-OH (spin adduct of OH˙) (a) and NO-Fe^2+^(MGD)_2_ (spin adduct of NO˙) (b).

It is generally realized that RONS of a PAS, such as atomic oxygen (O), the hydroxyl radical (OH˙), and the nitric oxide radical (NO˙), are related to bacterial inactivation. In previous studies, long-lived RONS, including hydrogen peroxide (H_2_O_2_), nitrite, and nitrate, were proven to contribute to the prolonged antibacterial effects of PASs (32). To better understand the mechanism of PASs, short-lived RONS (OH˙ and NO˙) were detected by electron spin resonance (ESR) spectroscopy in this study. As shown in Fig. 3, there were no signals in the water group. In contrast, 5,5-dimethyl-1-pyrroline-*N*-oxide (DMPO)-OH and NO-Fe^2+^(*N*-methyl-d-glucamine dithiocarbamate [MGD])_2_ signals were detected in PAS(H_2_O), PAS(NaCl), and PAS(H_2_O_2_), demonstrating that OH˙ and NO˙ were generated by plasma activation.

### Morphology studies of treated NDV.

**FIG 4:**
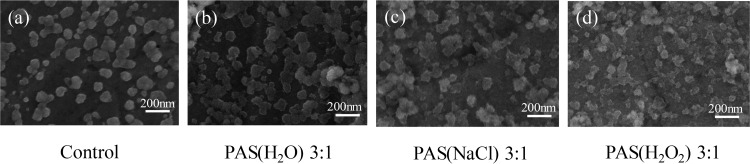
SEM images of NDV before and after PAS(H_2_O), PAS(NaCl), and PAS(H_2_O_2_) treatment at ratios of 3:1.

The morphological change was a pivotal factor, which could explain the inactivation mechanism of PASs. SEM was employed to verify the influence of PAS(H_2_O), PAS(NaCl), and PAS(H_2_O_2_) on the morphology of viral particles. As shown in Fig. 4a, the surface morphology of the virus in the control group was smooth and complete. However, after PAS treatment, the virus surface was shrunken and the structure was damaged, as shown in Fig. 4b, c, and d. The majority of virus particles showed a strong deformation of their external shape. The results demonstrated that RONS in PASs might result in changes of viral morphology and structure.

### Protein concentration of NDV.

**FIG 5:**
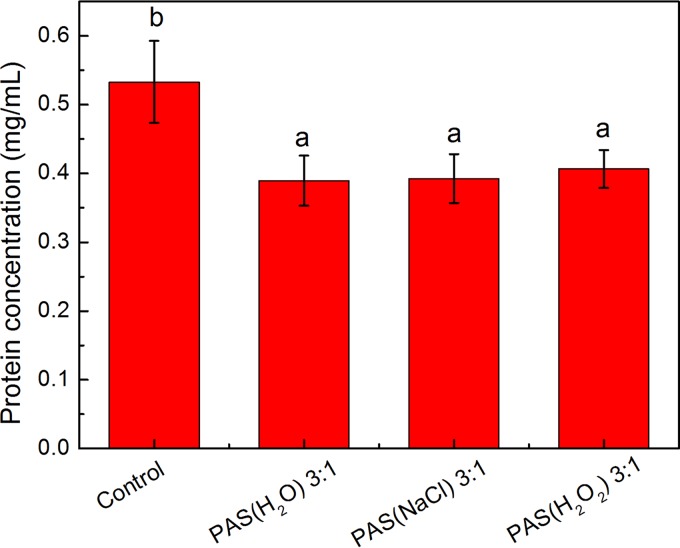
Protein concentrations after PAS(H_2_O), PAS(NaCl), and PAS(H_2_O_2_) treatments at ratios of 3:1. Bars labeled with different lowercase letters represent significant differences (*P* ≤ 0.05).

A rapid, sensitive, and versatile assay for protein detection involves using Coomassie brilliant blue G250. As shown in Fig. 5, the protein concentration of the untreated NDV-allantoic fluid was 0.53 mg/ml. After treatments with PAS(H_2_O), PAS(NaCl), and PAS(H_2_O_2_), the protein concentrations in the virus solutions were significantly decreased to 0.37, 0.38, and 0.42 mg/ml (*P* < 0.05). In addition, there was no significant difference among the protein concentrations of different PAS groups.

### Evaluation of RNA intensity.

**FIG 6:**
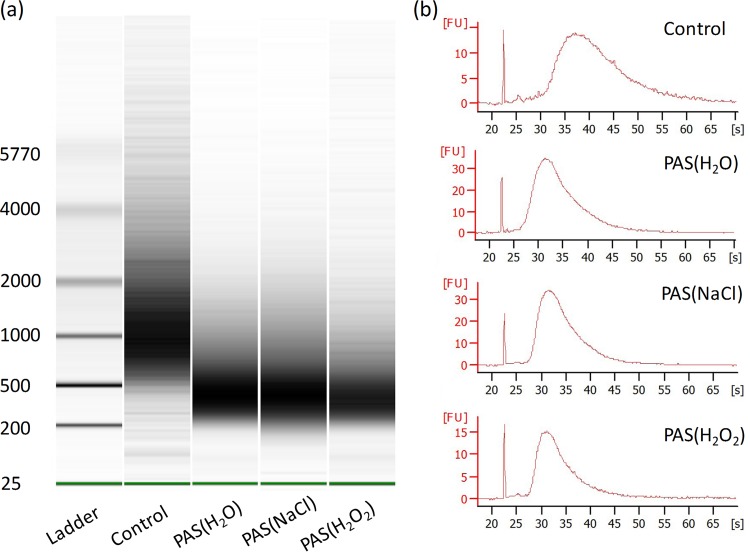
RNA integrity detection after PAS(H_2_O), PAS(NaCl), and PAS(H_2_O_2_) treatments at ratios of 3:1. (a) Virtual gel image of RNA generated by the Bioanalyzer software. (b) Fluorescence versus time data plot for different samples.

The application of an RNA intensity assay may bring more information about the mechanism of PAS inactivation and the interaction between PASs and living organisms. The Agilent 2100 bioanalyzer provides a platform for the electrophoresis of RNA on a disposable chip (33), which could evaluate the degree of degradation of RNA after PAS treatment. In this study, a virtual gel was visually analyzed and the fluorescence data were tabulated and graphed (Fig. 6a and b). The utilized RNA ladder was from 25 to 5,770 nucleotides (nt). The bioanalyzer analysis revealed the enrichment of RNA of the control group was for RNA of approximately 500 to 2,000 nt in length. After PAS(H_2_O), PAS(NaCl), and PAS(H_2_O_2_) treatments, the size distribution of RNA was 200 to 500 nt. The experimental evidence showed that the degradation of RNA after PAS exposure was detectable.

## DISCUSSION

The ELA and HA test results (Fig. 1) showed that different PASs could effectively inactivate NDV at a ratio of not less than 3:1. This further confirmed the effectiveness and completeness of PAS inactivation via three passages in chicken embryos (Table 1). To have a more in-depth understanding of the inactivation mechanism, follow-up detection and analysis, including of the physicochemical properties of PAS and the biological characteristics of virus after treatment, were used.

ORP was an indicator of the overall redox potential of the different solutions. After nonthermal plasma activation, there were obvious changes in different PASs, which might have an influence on the biological properties of the virus. A high ORP would destroy the surface proteins and the viral envelope, inactivate the viral enzymes, and cause damage to viral nucleic acids (34). In combination with the previous studies, we believed that ORP values in PAS(H_2_O), PAS(NaCl), and PAS(H_2_O_2_) may play a very important role in virus inactivation. Hydrogen peroxide, as a significant long-lived species in PAS, has attracted attention. The changes in H_2_O_2_ concentrations were consistent with the ORP results (Fig. 2a and b), which increased significantly after plasma activation. Several studies have also reported H_2_O_2_ in solution after plasma-activation. Naïtali et al. found that there were many long-lived active species, such as NO_3_^−^, NO_2_^−^ and H_2_O_2_, in plasma-activated water, which exerted a synergistic effect and were responsible for microbial decontamination (35). Golkowski et al. suggested that the disinfection efficacy of low-temperature plasma samples was enhanced by H_2_O_2_, which may be associated with the increased OH concentration (36) There were no significant differences among PAS(H_2_O), PAS(NaCl), and PAS(H_2_O_2_) on the basis of our results, which indicate that H_2_O_2_ in PAS(H_2_O_2_) may convert to other species during the process of plasma activation.

The increase in conductivity might be the interaction between the active substance and water molecules (37). Meanwhile, it has been reported that the increase in conductivity might promote the photolysis of H_2_O_2_ and increase the yield of OH (38). The conductivity of PAS(NaCl) was higher than those of the other treatment groups (Fig. 2c). A possible explanation is that the following reactions occurred.

(1) OH+Cl−·→Cl·+OH−

(2) $${\text{Cl}}^{\cdot }+{\text{Cl}}^{\cdot }\to {\text{Cl}}_{2}$$

(3) $${\text{Cl}}_{2}+{\text{H}}_{2}\text{O}\to \text{HCl}+\text{HClO}$$

(4) $$2\text{HClO}+{\text{H}}_{2}{\text{O}}_{2}\to 2{\text{Cl}}^{-}+{2\text{O}}_{2}+{4\text{H}}^{+}$$

When plasma jetting was performed in a 0.9% NaCl solution, Cl^−^ could be converted to a chlorine radical and form HClO according to reaction 3. HClO is known to be the major bactericidal agent in electrochemical inactivation (39). Subsequently, HClO reacts with H_2_O_2_ to produce H^+^ (40, 41). As a result of the higher ionic mobility of H^+^ than other ions, the electrical conductivity in PAS(NaCl) was higher than in PAS(H_2_O) and PAS(H_2_O_2_). In consideration of ELA and HA test results, the electrical conductivity of PAS might be responsible for the inactivation of NDV, which was significantly higher than in the control group. Moreover, the role of conductivity in plasma-activated water sterilization has been confirmed (37). On the other hand, the electrical conductivities of different PASs made a great difference. However, there was little difference in the inactivation efficacies of different PASs, which needs further study.

It is generally agreed that the combination of a high ORP and low pH is responsible for the inactivation ability of PASs, which is in agreement with our results. Previous studies have reported that the reactive species effectively inactivate the bacteria at low pH (42). Simultaneously, it was proved that the ORP alters the redox potential and initiates the peroxidation of fatty acids in the cell membrane under acidic conditions (43, 44).

In addition to some long-lived species, such as H_2_O_2_, nitrite, and nitrates, several short-lived RONS (OH˙ and NO˙) were detected by ESR spectroscopy in this study (Fig. 3). It was speculated that the production of radicals was via the following reactions (45).

(5) $${\text{H}}_{2}\text{O}+{\text{e}}^{-}\to \text{OH}+\text{H}+{\text{e}}^{-}$$

(6) $${\text{N}}_{2}+{\text{e}}^{-}\to 2\text{N}+{\text{e}}^{-}$$

(7) $${\text{O}}_{2}+{\text{e}}^{-}\to 2\text{O}+{\text{e}}^{-}$$

(8) N+O→NO

OH˙ can easily attack unsaturated fatty acids on the cell membrane and interfere with large intracellular molecules (46). NO˙ can change ion currents, cause DNA mutation, and inhibit DNA repair and enzyme activities, resulting in cellular necrosis (47). Therefore, OH˙ and NO˙ existing in PASs may be the vital factors responsible for the virus inactivation.

It was observed that certain proteins of the virus were damaged and degraded after PAS treatment (Fig. 5), which was associated with the morphological change of the virus (Fig. 4). According to a previous study, Wu et al. proposed that nonthermal plasma can destroy surface proteins and related RNA genes of MS2, leading to viral inactivation and a loss of infectivity (48), which depend on the power level and the working gas. These active species could act at the protein level and cause protein peroxidation and degradation, thereby contributing to the destruction of the capsid. On the basis of the results shown in Fig. 6, it was suggested that PASs could result in the breakage of RNA, which was most likely attributed to RONS in the PASs. These reactive species may attack the RNA sugar phosphate backbone. Yasuda et al. also proposed that nonthermal plasma inactivates bacteriophage by affecting the viral coat protein and causing damage to the DNA (49).

Altogether, the inactivation mechanisms of PASs against virus can be speculated, including the following steps (1). Nonthermal plasma could produce a large number of free radicals in the PAS, leading to high ORP and electrical conductivity (2). OH˙, NO˙, and H_2_O_2_, as representative of short-lived and long-lived species in the PAS, are the major species generated by nonthermal plasma. They could react with carbohydrates and initiate lipid peroxidation and cross-linking of the fatty acid side chains, resulting in alterations of the chemical bonds and molecular structure (3). RONS could induce oxidative stress in the virus. On the one hand, they act at the protein level, causing protein peroxidation and inducing the destruction of the virus envelope, ultimately resulting in changes to the viral morphology. On the other hand, they can damage viral nucleic acids encoding enzymes, contributing to reduced gene expression and the elimination of virus replication, thereby leading to virus inactivation. These findings suggest that the treated fluid may carry RONS, which maintain the inactivation properties. However, it is not yet fully known which exact species and products are generated in these fluids (i.e., deionized water, NaCl, and H_2_O_2_), which need authentication by several techniques for detailed chemical characterizations.

### Conclusion.

In summary, this study indicates that a PAS has the potential to inactivate NDV. PAS(H_2_O), PAS(NaCl), and PAS(H_2_O_2_) as antiviral solutions can completely inactivate NDV at an appropriate ratio, which was verified by ELA and HA tests. These lay a foundation for the application of PASs to resolve the issues of public health and environmental sanitation. The possible mechanism of PAS inactivation against NDV is that RONS, including short-lived OH˙ and NO˙ and long-lived H_2_O_2_ detected in PAS, might change viral morphology, destroy the viral RNA structure, and degrade viral protein, consequently resulting in virus inactivation. Therefore, the application of PASs, which are environmentally friendly, can be a promising alternative strategy in poultry industries. Further studies are required to verify whether these changes are sufficient to disrupt virus functions and to further shed light on the determinant factors for virus inactivation by PASs.

## MATERIALS AND METHODS

### Plasma microjet device and PAS generation.

**FIG 7:**
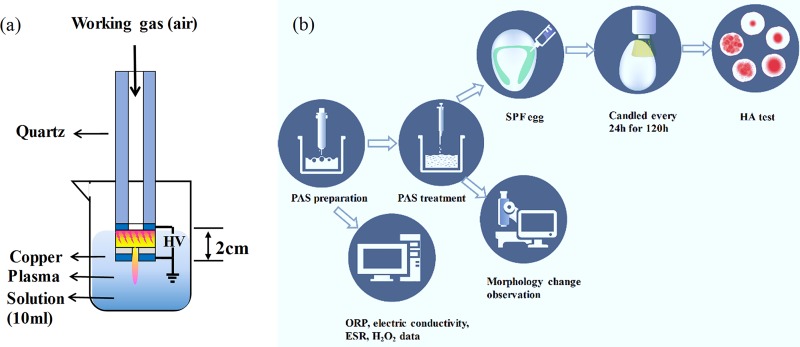
(a) Schematic diagram of the PMJ system and the process of PAS generation. (b) Experimental arrangement description. SPF, specific pathogen free; ELA, embryo lethality assay; HA test, hemagglutination test; ESR, electron spin resonance; ORP, oxidation-reduction potential; NDV, Newcastle disease virus.

The air plasma generator, schematically illustrated in Fig. 7a, was designed on the basis of a dielectric barrier structure with hollow electrodes (HEDBS). The device was composed of a copper electrode and dielectric quartz. The working gas was air with a 260-liter/h flow rate, which was injected into the quartz tube. The high-voltage electrode and a power source (20 kHz) were connected. Homogeneous plasma was produced in the discharge gap of 0.5 mm and was ejected through the end outlet of 0.5 mm, reaching 7 mm long. The specific details about the structure have been described by Yu et al. (50).

As shown in Fig. 7b, the PASs were generated by plasma activation beneath the solution surfaces. The distance between the liquid surface and the plasma microjet (PMJ) end was 20 mm. The 10-ml sterile distilled water, 0.9% NaCl, and 0.3% H_2_O_2_ solutions were activated by plasma for 10 min to obtain the PASs, defined as PAS(H_2_O), PAS(NaCl), and PAS(H_2_O_2_), respectively, which were freshly prepared for inactivating NDV.

### PAS treatment.

NDV (LaSota strain) was propagated in 10-day-old specific-pathogen-free (SPF) chicken embryos. The embryos that died within 24 h were discarded, and the allantoic fluids were harvested, centrifuged at 1,000 × *g*, and stored at −20°C before use. Fifty percent egg infectious dose (EID_50_) titers were calculated by the method of Reed and Muench after serial titration in eggs (51, 52). To assess the inactivation ability of the PASs, the allantoic fluids containing NDV were treated with PAS(H_2_O), PAS(NaCl), and PAS (H_2_O_2_) at ratios of 1:3, 1:5, and 1:9, respectively, for 30 min. The NDV-allantoic fluid without PAS treatment was used as the control.

### Embryo lethality assay.

Viruses treated by PAS(H_2_O), PAS(NaCl), and PAS(H_2_O_2_) at different ratios and the control sample were inoculated into 10-day-old SPF chicken embryos via an intra-allantoic route, with five embryos in each group. Every embryo was injected with 0.1 ml of 10^8.3^ EID_50_ NDV treated by PAS. According to previous studies (53–55), the eggs were candled every 24 h, and the embryos that died within 24 h were discarded. The death number beyond 24 h was recorded until 120 h, which was summarized as the ELA results. The SPF chicken embryos were supplied by Beijing Merial Vital Laboratory Animal Technology Co., Ltd.

### HA test.

The HA assays conform to OIE standard procedures (56). To perform the HA test, 50 μl of phosphate-buffered saline (PBS) was added to every well of a 96-well plate. Then, 50 μl of the samples was added to the first column of the plates and mixed thoroughly. Subsequently, 50 μl of the mixture in the first column was added to the second column accordingly and mixed again. In accordance with the above procedure, the samples were constantly diluted until they were added to the 11th column. After mixing, 50 μl of the mixture in the 11th column was discarded. Finally, 50 μl of 1% erythrocytes was added to every well. After slight oscillation, the plates were placed for 25 min at 37°C. The HA titer was read as log_2_ of the highest dilution of antigen giving complete HA (no streaming).

### Virus blind passage assay.

To verify whether the virulence of NDV treated by PASs could be recovered, the virus after PAS treatment was injected into 10-day-old SPF chicken embryos. Inoculated eggs were incubated at 37°C and candled every 24 h. Embryos that died within 24 h were discarded. The remaining embryos were chilled after 120 h of inoculation. Subsequently, the allantoic fluids were extracted from the corresponding embryos, and 0.1 ml of the collected allantoic fluid was injected into the second group of 10-day-old chicken embryos for 120 h of observation. In the same way, allantoic fluid was obtained from the embryos and was inoculated in the third group of 10-day-old chicken embryos. After three passages, the allantoic fluids were harvested and used for the determination of virus titers by ELAs and HA tests.

### Detection of ORP, H_2_O_2_ concentration, electrical conductivity, and pH.

ORP was an indicator of the general level of reactive oxygen species (57). The ORPs of the different PASs were determined using an ORP probe (LE501 and LE510; Mettler Toledo, USA) according to the operation procedure. The experiment was performed randomly three times. As the representative long-lived species, the H_2_O_2_ concentration in PAS samples was quantified by a hydrogen peroxide assay kit (Beyotime, Jiangsu, China). After PAS(H_2_O), PAS(NaCl), and PAS(H_2_O_2_) were prepared, the electrical conductivities of PAS(H_2_O), PAS(NaCl), and PAS(H_2_O_2_) were immediately measured with an electrical conductivity meter (DDB-303A). pH values were recorded by a microprocessor pH meter (Mettler-Toledo LE438).

### Electron spin resonance spectroscopy.

Electron spin resonance (ESR) spectroscopy, which is based on the interaction of unpaired electron spins with an external magnetic field, was employed to measure the significant reactive oxygen nitrogen species (RONS), the hydroxyl radical (OH˙), and the nitric oxide radical (NO˙) (58). In view of the short lifetimes of OH˙ (59) and NO˙ (60), after PAS generation, 20 μl of 5,5-dimethyl-1-pyrroline-N-oxide (DMPO) (0.8 M; Sigma-Aldrich, USA) was added immediately to 20 μl of each PAS and mixed thoroughly to spin trap OH˙. Two hundred fifty microliters of *N*-methyl-d-glucamine dithiocarbamate (MGD) (1.0 M, 99%; J&K Scientific Ltd. China) and 250 μl Fe^2+^ (0.3 M) were added immediately to 0.5-ml samples to spin trap NO˙ via forming a longer-lived spin adduct complex, NO-Fe^2+^(MGD)_2_. The final product was taken up by a capillary and detected in the resonator cavity of an ESR spectrometer (ER-200D-SRC/E-500; Bruker Ltd., Germany) operated at room temperature.

### Scanning electron microscopy.

A scanning electron microscope (S-4800; Hitachi, Japan) was used to observe the overall morphological changes of viruses treated with PAS(H_2_O), PAS(NaCl), and PAS(H_2_O_2_) in the experiment. Viruses were isolated with differential centrifugation. The viral allantoic fluids were centrifuged at a low speed of 1,000 × *g*. After the removal of the precipitates, the supernatants were centrifuged at 20,000 × *g* and fixed with 2.5% glutaraldehyde overnight at 4°C. Then, the samples were dehydrated through a graded ethanol series (30%, 50%, 70%, 90%, and 100% ethanol; 10 min in each solution) at room temperature. Finally, the overall morphological changes of the viruses were observed by SEM (61).

### Protein concentration of NDV.

Coomassie brilliant blue G250 is an organic dye that, combined with the protein alkaline amino acids and aromatic amino acid residues in the dilute acid solution, forms a blue complex (62, 63). In this study, PAS(H_2_O), PAS(NaCl), PAS(H_2_O_2_), and viral allantoic fluids interacted at a ratio of 3:1 and were detected by a Bradford protein assay kit (Beyotime, Jiangsu, China). In accordance with the procedures, 200 μl of the G250 dye solution and 20 μl of the samples were added to the 96-well plate for 3 to 5 min at room temperature. The protein concentrations were calculated by the absorbance values at 595 nm on a SPECTROstar Omega plate reader (BMG, Germany).

### Evaluation of RNA intensity.

It is essential to determine the quality of RNA after PAS treatment. The NDV-allantoic fluid supernatants were first filtered through 0.45-μm filters (Millipore, USA). Viral RNAs were extracted using an All Prep DNA/RNA minikit or a QIAamp MinElute virus spin kit (Qiagen, Germany). The concentrations of extracted RNA were approximately 50 ng/μl, and the samples were stored at −80°C before use. To assess the RNA integrity, the Agilent 2100 bioanalyzer was used in combination with the RNA 6000 Nano LabChip kit (Agilent Technologies) (64–66). Briefly, 9 μl of the gel-dye mixture was added to the appropriate well. Subsequently, the ladder and sample wells were loaded with 5 μl of the marker mixture and 1 μl of either the ladder or the sample. After mixing by vortexing, the chips were immediately inserted into the bioanalyzer and the experiment as carried out according to the instructions of the manufacturer. All experiments were performed using Agilent Biosizing software (version A.01.10). All samples were diluted in nuclease-free non-DEPC-treated water.

### Statistical analysis.

All data were obtained from at least three replicate experiments (*n* ≥ 3). Values from all experiments are expressed as the means and standard deviations (SDs). Statistical analyses were conducted using SPSS statistical package 17.0 (SPSS Inc., USA). An analysis of variance (ANOVA) was performed to assess the physicochemical properties of PAS(H_2_O), PAS(NaCl), and PAS(H_2_O_2_), including ORP, electrical conductivity, and H_2_O_2_ concentration, and to evaluate the effects of different PASs on the protein concentrations of NDV-allantoic fluid. Significant differences between mean values were identified by the Student-Newman-Keuls multiple range test with a confidence level at a *P* value of ≤0.05. Furthermore, the paired-sample *t* test was applied to compare the inactivation efficacies of different PASs against NDV.

## References

[1] Seal BS, King DJ, Sellers HS 2000 The avian response to Newcastle disease virus. Dev Comp Immunol 24:257–268. doi:10.1016/S0145-305X(99)00077-4. 10717292

[2] Alexander D 2000 Newcastle disease, other avian paramyxoviruses, and pneumovirus infections, p 63–99. *In* Saif YM, Glisson JR, Fadly AM, McDougald LR, Swayne D (ed), Diseases of poultry. Iowa State University Press, Ames, IA.

[3] Ganar K, Das M, Sinha S, Kumar S 2014 Newcastle disease virus: current status and our understanding. Virus Res 184:71–81. doi:10.1016/j.virusres.2014.02.016. 24589707 PMC7127793

[4] World Bank. 2011 World livestock disease atlas: a quantitative analysis of global animal health data (2006–2009). The World Bank, Washington, DC http://documents.worldbank.org/curated/en/323671468179364909/pdf/668590WP00PUBL00Livestock0Atlas0web.pdf.

[5] Kaleta EF, Baldauf C 1988 Newcastle disease in free-living and pet birds, p 197–246. *In* Alexander DJ (ed), Newcastle disease. Springer, Boston, MA.

[6] Kim LM, King DJ, Guzman H, Tesh RB, Travassos da Rosa APA, Bueno R, Dennett JA, Afonso CL 2008 Biological and phylogenetic characterization of pigeon paramyxovirus serotype 1 circulating in wild North American pigeons and doves. J Clin Microbiol 46:3303–3310. doi:10.1128/JCM.00644-08. 18716227 PMC2566108

[7] Pchelkina IP, Manin TB, Kolosov SN, Starov SK, Andriyasov AV, Chvala IA, Drygin VV, Yu Q, Miller PJ, Suarez DL 2013 Characteristics of pigeon paramyxovirus serotype-1 isolates (PPMV-1) from the Russian Federation from 2001 to 2009. Avian Dis 57:2–7. doi:10.1637/10246-051112-Reg.1. 23678722

[8] Alexander DJ 2000 Newcastle disease and other avian paramyxoviruses. Rev Sci Tech 19:443–462. doi:10.20506/rst.19.2.1231. 10935273

[9] Kapczynski DR, Afonso CL, Miller PJ 2013 Immune responses of poultry to Newcastle disease virus. Dev Comp Immunol 41:447–453. doi:10.1016/j.dci.2013.04.012. 23623955

[10] Swayne DE, King DJ 2003 Avian influenza and Newcastle disease. J Am Vet Med Assoc 222:1534–1540. doi:10.2460/javma.2003.222.1534. 12784958

[11] Laroussi M 2005 Low temperature plasma-based sterilization: overview and state-of-the-art. Plasma Process Polym 2:391–400. doi:10.1002/ppap.200400078.

[12] Niemira BA 2012 Cold plasma decontamination of foods. Annu Rev Food Sci Technol 3:125–142. doi:10.1146/annurev-food-022811-101132. 22149075

[13] Pankaj SK, Bueno-Ferrer C, Misra NN, Milosavljević V, O'Donnell CP, Bourke P, Keener KM, Cullen PJ 2014 Applications of cold plasma technology in food packaging. Trends Food Sci Technol 35:5–17. doi:10.1016/j.tifs.2013.10.009.

[14] Pan J, Yang X, Sun K, Wang J, Sun P, Wu H, Becker KH, Zhu W, Zhang J, Fang J 2013 Tooth bleaching using low concentrations of hydrogen peroxide in the presence of a nonthermal plasma jet. IEEE Trans Plasma Sci 41:325–329. doi:10.1109/TPS.2012.2233753.

[15] Ma R, Wang G, Tian Y, Wang K, Zhang J, Fang J 2015 Non-thermal plasma-activated water inactivation of food-borne pathogen on fresh produce. J Hazard Mater 300:643–651. doi:10.1016/j.jhazmat.2015.07.061. 26282219

[16] Xu Y, Tian Y, Ma R, Liu Q, Zhang J 2016 Effect of plasma activated water on the postharvest quality of button mushrooms, *Agaricus bisporus*. Food Chem 197:436–444. doi:10.1016/j.foodchem.2015.10.144. 26616972

[17] Oehmigen K, Hähnel M, Brandenburg R, Wilke C, Weltmann KD, von Woedtke T 2010 The role of acidification for antimicrobial activity of atmospheric pressure plasma in liquids. Plasma Process Polym 7:250–257. doi:10.1002/ppap.200900077.

[18] Kamgang-Youbi G, Herry JM, Meylheuc T, Brisset JL, Bellon-Fontaine MN, Doubla A, Naïtali M 2009 Microbial inactivation using plasma-activated water obtained by gliding electric discharges. Lett Appl Microbiol 48:13–18. doi:10.1111/j.1472-765X.2008.02476.x. 19170858

[19] Burlica R, Grim RG, Shih KY, Balkwill D, Locke BR 2010 Bacteria inactivation using low power pulsed gliding arc discharges with water spray. Plasma Process Polym 7:640–649. doi:10.1002/ppap.200900183.

[20] Traylor MJ, Pavlovich MJ, Karim S, Hait P, Sakiyama Y, Clark DS, Graves DB 2011 Long-term antibacterial efficacy of air plasma-activated water. J Phys D Appl Phys 44:472001. doi:10.1088/0022-3727/44/47/472001.

[21] van Gils CAJ, Hofmann S, Boekema BKHL, Brandenburg R, Bruggeman PJ 2013 Mechanisms of bacterial inactivation in the liquid phase induced by a remote RF cold atmospheric pressure plasma jet. J Phys D Appl Phys 46:175203. doi:10.1088/0022-3727/46/17/175203.

[22] Dobrynin D, Fridman G, Friedman G, Fridman A 2009 Physical and biological mechanisms of direct plasma interaction with living tissue. New J Phys 11:115020. doi:10.1088/1367-2630/11/11/115020.

[23] Oehmigen K, Winter J, Hähnel M, Wilke C, Brandenburg R, Weltmann K-D, von Woedtke T 2011 Estimation of possible mechanisms of *Escherichia coli* inactivation by plasma treated sodium chloride solution. Plasma Process Polym 8:904–913. doi:10.1002/ppap.201000099.

[24] Zhang Q, Liang Y, Feng H, Ma R, Tian Y, Zhang J, Fang J 2013 A study of oxidative stress induced by non-thermal plasma-activated water for bacterial damage. Appl Phys Lett 102:203701. doi:10.1063/1.4807133.

[25] Pavlovich MJ, Chang HW, Sakiyama Y, Clark DS, Graves DB 2013 Ozone correlates with antibacterial effects from indirect air dielectric barrier discharge treatment of water. J Phys D Appl Phys 46:145202. doi:10.1088/0022-3727/46/14/145202.

[26] Mohades S, Laroussi M, Sears J, Barekzi N, Razavi H 2015 Evaluation of the effects of a plasma activated medium on cancer cells. Phys Plasmas 22:122001. doi:10.1063/1.4933367.

[27] Park DP, Davis K, Gilani S, Alonzo CA, Dobrynin D, Friedman G, Fridman A, Rabinovich A, Fridman G 2013 Reactive nitrogen species produced in water by non-equilibrium plasma increase plant growth rate and nutritional yield. Curr Appl Phys 13:S19–S29. doi:10.1016/j.cap.2012.12.019.

[28] Alshraiedeh NH, Alkawareek MY, Gorman SP, Graham WG, Gilmore BF 2013 Atmospheric pressure, nonthermal plasma inactivation of MS2 bacteriophage: effect of oxygen concentration on virucidal activity. J Appl Microbiol 115:1420–1426. doi:10.1111/jam.12331. 23957472

[29] Alekseev O, Donovan K, Limonnik V, Azizkhan-Clifford J 2014 Nonthermal dielectric barrier discharge (DBD) plasma suppresses herpes simplex virus type 1 (HSV-1) replication in corneal epithelium. Transl Vis Sci Technol 3:2. doi:10.1167/tvst.3.2.2. PMC3969218 24757592

[30] Zimmermann JL, Dumler K, Shimizu T, Morfill GE, Wolf A, Boxhammer V, Schlegel J, Gansbacher B, Anton M 2011 Effects of cold atmospheric plasmas on adenoviruses in solution. J Phys D Appl Phys 44:505201. doi:10.1088/0022-3727/44/50/505201.

[31] Aboubakr HA, Williams P, Gangal U, Youssef MM, El-Sohaimy SAA, Bruggeman PJ, Goyal SM 2015 Virucidal effect of cold atmospheric gaseous plasma on feline calicivirus, a surrogate for human norovirus. Appl Environ Microbiol 81:3612–3622. doi:10.1128/AEM.00054-15. 25795667 PMC4421051

[32] Shen J, Tian Y, Li Y, Ma R, Zhang Q, Zhang J, Fang J 2016 Bactericidal dffects against *S. aureus* and physicochemical properties of plasma activated water stored at different temperatures. Sci Rep 6:28505. doi:10.1038/srep28505. 27346695 PMC4921907

[33] Crocitto LE, Korns D, Kretzner L, Shevchuk T, Blair SL, Wilson TG, Ramin SA, Kawachi MH, Smith SS 2004 Prostate cancer molecular markers GSTP1 and hTERT in expressed prostatic secretions as predictors of biopsy results. Urology 64:821–825. doi:10.1016/j.urology.2004.05.007. 15491741

[34] Tagawa M, Yamaguchi T, Yokosuka O, Matsutani S, Maeda T, Saisho H 2000 Inactivation of a hepadnavirus by electrolysed acid water. J Antimicrob Chemother 46:363–368. doi:10.1093/jac/46.3.363. 10980161

[35] Naïtali M, Kamgang-Youbi G, Herry J-M, Bellon-Fontaine M-N, Brisset J-L 2010 Combined effects of long-living chemical species during microbial inactivation using atmospheric plasma-treated water. Appl Environ Microbiol 76:7662–7664. doi:10.1128/AEM.01615-10. 20889799 PMC2976197

[36] Golkowski M, Golkowski C, Leszczynski J, Plimpton SR, Maslowski P, Foltynowicz A, Ye J, McCollister B 2012 Hydrogen-peroxide-enhanced nonthermal plasma effluent for biomedical applications. IEEE Trans Plasma Sci 40:1984–1991. doi:10.1109/TPS.2012.2200910.

[37] Maeda Y, Igura N, Shimoda M, Hayakawa I 2003 Bactericidal effect of atmospheric gas plasma on *Escherichia coli* K-12. Int J Food Sci Technol 38:889–892. doi:10.1046/j.1365-2621.2003.00746.x.

[38] Lukes P, Clupek M, Babicky V, Sunka P 2008 Ultraviolet radiation from the pulsed corona discharge in water. Plasma Sources Sci Technol 17:024012. doi:10.1088/0963-0252/17/2/024012.

[39] Sakiyama Y, Tomai T, Miyano M, Graves DB 2009 Disinfection of *E. coli* by nonthermal microplasma electrolysis in normal saline solution. Appl Phys Lett 94:161501. doi:10.1063/1.3122148.

[40] Lukes P, Appleton AT, Locke BR 2004 Hydrogen peroxide and ozone formation in hybrid gas-liquid electrical discharge reactors. IEEE Trans Ind Appl 40:60–67. doi:10.1109/TIA.2003.821799.

[41] Aubry JM 1985 Search for singlet oxygen in the decomposition of hydrogen peroxide by mineral compounds in aqueous solutions. J Am Chem Soc 107:5844–5849. doi:10.1021/ja00307a002.

[42] Ikawa S, Kitano K, Hamaguchi S 2010 Effects of pH on bacterial inactivation in aqueous solutions due to low-temperature atmospheric pressure plasma application. Plasma Process Polym 7:33–42. doi:10.1002/ppap.200900090.

[43] Kim SJ, Chung TH, Bae SH, Leem SH 2009 Bacterial inactivation using atmospheric pressure single pin electrode microplasma jet with a ground ring. Appl Phys Lett 94:141502. doi:10.1063/1.3114407.

[44] Bielski BHJ, Cabelli DE, Arudi RL, Ross AB 1985 Reactivity of HO_2_/O^−^_2_ radicals in aqueous solution. J Phys Chem Ref Data 14:1041–1100. doi:10.1063/1.555739.

[45] Bruggeman P, Schram DC 2010 On OH production in water containing atmospheric pressure plasmas. Plasma Sources Sci Technol 19:045025. doi:10.1088/0963-0252/19/4/045025.

[46] Cheeseman KH, Slater TF 1993 An introduction to free radical biochemistry. Br Med Bull 49:481–493. doi:10.1093/oxfordjournals.bmb.a072625. 8221017

[47] Moisan M, Barbeau J, Moreau S, Pelletier J, Tabrizian M, Yahia LH 2001 Low-temperature sterilization using gas plasmas: a review of the experiments and an analysis of the inactivation mechanisms. Int J Pharm 226:1–21. doi:10.1016/S0378-5173(01)00752-9. 11532565

[48] Wu Y, Liang Y, Wei K, Li W, Yao M, Zhang J, Grinshpun SA 2015 MS2 virus inactivation by atmospheric-pressure cold plasma using different gas carriers and power levels. Appl Environ Microbiol 81:996–1002. doi:10.1128/AEM.03322-14. 25416775 PMC4292470

[49] Yasuda H, Miura T, Kurita H, Takashima K, Mizuno A 2010 Biological evaluation of DNA damage in bacteriophages inactivated by atmospheric pressure cold plasma. Plasma Process Polym 7:301–308. doi:10.1002/ppap.200900088.

[50] Yu S, Chen Q, Liu J, Wang K, Jiang Z, Sun Z, Zhang J, Fang J 2015 Dielectric barrier structure with hollow electrodes and its recoil effect. Appl Phys Lett 106:244101. doi:10.1063/1.4922395.

[51] Wise MG, Suarez DL, Seal BS, Pedersen JC, Senne DA, King DJ, Kapczynski DR, Spackman E 2004 Development of a real-time reverse-transcription PCR for detection of Newcastle disease virus RNA in clinical samples. J Clin Microbiol 42:329–338. doi:10.1128/JCM.42.1.329-338.2004. 14715773 PMC321685

[52] Reed LJ, Muench H 1938 A simple method of estimating fifty per cent endpoints. Am J Epidemiol 27:493–497. doi:10.1093/oxfordjournals.aje.a118408.

[53] Desingu PA, Singh SD, Dhama K, Kumar ORV, Singh R, Singh RK 2015 A rapid method of accurate detection and differentiation of Newcastle disease virus pathotypes by demonstrating multiple bands in degenerate primer based nested RT-PCR. J Virol Methods 212:47–52. doi:10.1016/j.jviromet.2014.11.005. 25449112

[54] Goyal SM, Anantharaman S, Ramakrishnan MA, Sajja S, Kim SW, Stanley NJ, Farnsworth JE, Kuehn TH, Raynor PC 2011 Detection of viruses in used ventilation filters from two large public buildings. Am J Infect Control 39:e30–e38. doi:10.1016/j.ajic.2010.10.036. 21549446 PMC7132662

[55] Westbury HA 1979 Newcastle disease virus–some properties of Australian strains. Avian Dis 23:564–570. doi:10.2307/1589731. 526199

[56] Office International des Epizooties (OIE). 2008 Manual of diagnostic tests and vaccines for terrestrial animals (mammals, birds and bees), 6th ed, vol 1 Office International des Epizooties (OIE), Paris, France.

[57] McFerson LL 1993 Understanding ORP's role in the disinfection process. Water Eng Manage 140:29–31.

[58] Halliwell B 2006 Reactive species and antioxidants. Redox biology is a fundamental theme of aerobic life. Plant Physiol 141:312–322. doi:10.1104/pp.106.077073. 16760481 PMC1475431

[59] Sun P, Wu H, Bai N, Zhou H, Wang R, Feng H, Zhu W, Zhang J, Fang J 2012 Inactivation of *Bacillus subtilis* spores in water by a direct-current, cold atmospheric-pressure air plasma microjet. Plasma Process Polym 9:157–164. doi:10.1002/ppap.201100041.

[60] Palmer RMJ, Ferrige AG, Moncada S 1987 Nitric oxide release accounts for the biological activity of endothelium-derived relaxing factor. Nature 327:524–526. doi:10.1038/327524a0. 3495737

[61] Wang G, Zhu R, Yang L, Wang K, Zhang Q, Su X, Yang B, Zhang J, Fang J 2016 Non-thermal plasma for inactivated-vaccine preparation. Vaccine 34:1126–1132. doi:10.1016/j.vaccine.2015.10.099. 26529075

[62] Kruger NJ 1994 The Bradford method for protein quantitation, p 9–15. *In* Walker JM (ed), Basic protein and peptide protocols. Humana Press, Totowa, NJ.

[63] Bradford MM 1976 A rapid and sensitive method for the quantitation of microgram quantities of protein utilizing the principle of protein-dye binding. Anal Biochem 72:248–254. doi:10.1016/0003-2697(76)90527-3. 942051

[64] Nachamkin I, Panaro NJ, Li M, Ung H, Yuen PK, Kricka LJ, Wilding P 2001 Agilent 2100 bioanalyzer for restriction fragment length polymorphism analysis of the *Campylobacter jejuni* flagellin gene. J Clin Microbiol 39:754–757. doi:10.1128/JCM.39.2.754-757.2001. 11158144 PMC87813

[65] Sun W, Zhao C, Li Y, Wang L, Nie G, Peng J, Wang A, Zhang P, Tian W, Li Q, Song J, Wang C, Xu X, Tian Y, Zhao D, Xu Z, Zhong G, Han B, Ling S, Chang Y-Z, Li Y 2016 Osteoclast-derived microRNA-containing exosomes selectively inhibit osteoblast activity. Cell Discov 2:16015. doi:10.1038/celldisc.2016.15. 27462462 PMC4886818

[66] Lu C-Y, Tso D-J, Yang T, Jong Y-J, Wei Y-H 2002 Detection of DNA mutations associated with mitochondrial diseases by Agilent 2100 bioanalyzer. Clin Chim Acta 318:97–105. doi:10.1016/S0009-8981(01)00809-9. 11880118

